# *Mycobacterium tuberculosis*-macrophage interaction: Molecular updates

**DOI:** 10.3389/fcimb.2023.1062963

**Published:** 2023-03-03

**Authors:** Haotian Bo, Ulrich Aymard Ekomi Moure, Yuanmiao Yang, Jun Pan, Li Li, Miao Wang, Xiaoxue Ke, Hongjuan Cui

**Affiliations:** ^1^ State Key Laboratory of Silkworm Genome Biology, Southwest University, Chongqing, China; ^2^ The Ninth People's Hospital of Chongqing, Affiliated Hospital of Southwest University, Chongqing, China; ^3^ Cancer Center, Medical Research Institute, Southwest University, Chongqing, China; ^4^ Jinfeng Laboratory, Chongqing, China

**Keywords:** intracellular pathogen, *Mycobacterium tuberculosis*, host macrophage, molecular interaction, immune control, immune evasion, tuberculosis control

## Abstract

*Mycobacterium tuberculosis* (Mtb), the causative agent of Tuberculosis (TB), remains a pathogen of great interest on a global scale. This airborne pathogen affects the lungs, where it interacts with macrophages. Acidic pH, oxidative and nitrosative stressors, and food restrictions make the macrophage’s internal milieu unfriendly to foreign bodies. Mtb subverts the host immune system and causes infection due to its genetic arsenal and secreted effector proteins. *In vivo* and *in vitro* research have examined Mtb-host macrophage interaction. This interaction is a crucial stage in Mtb infection because lung macrophages are the first immune cells Mtb encounters in the host. This review summarizes Mtb effectors that interact with macrophages. It also examines how macrophages control and eliminate Mtb and how Mtb manipulates macrophage defense mechanisms for its own survival. Understanding these mechanisms is crucial for TB prevention, diagnosis, and treatment.

## 1 Introduction

The host mucosal barrier is formed by macrophages, epithelial lining, inflammation responses, and released soluble substances (Graves and Milovanova, 2019). Macrophages are programmed to detect invading pathogens, activate microbicidal mechanisms, and coordinate the subsequent immune responses (Ravesloot-Chávez et al., 2021). Macrophages are located in every part of the body, and in the lungs, two main populations of macrophages have been identified, including alveolar macrophages (tissue-resident alveolar macrophages and monocyte-derived alveolar macrophages), and interstitial macrophages (Hou et al., 2021). The first group, on the inner surface of the lungs, accounts for 55% of lung immune cells (Hume et al., 2020) and suppresses intracellular pathogens, including Mtb (Hou et al., 2021).

Mtb is an obligate intracellular pathogen affecting about 10 million people each year among which 1.5 million lost their lives, and these statistics have been worsen with the new coronavirus disease (COVID-19) pandemic that has reversed gains and set back the fight against TB by several years (Global Tuberculosis Report, 2021). This airborne pathogen enters the lungs through the respiratory tract and firstly attacks aveolar macrophages. This Mtb-host macrophage interaction is a critical step for Mtb to successfully establish the infection. Mtb infection process can be randomly resumed as follows: 1) Mtb attachment by pathogen-associated molecular patterns (PAMPs); 2) Mtb identification by host macrophage pattern recognition receptors (PRRs) (**Table 1**); and 3) macrophage stimulation and activation of intracellular cascades and signaling pathways (BoseDasgupta and Pieters, 2018; Yassine et al., 2021). Mtb's intracellular interaction with macrophages is complex and challenging because of the hostile intracellular macrophage milieu (ROS and RNS production, low pH, food depletion, etc.) (Weiss and Schaible, 2015). Despite macrophages' impassable barrier, Mtb can evade host macrophage defenses *via* evolved escaping strategies to successfully create infection (Flentie et al., 2016; Chandra et al., 2022). This escape ability affects TB control, including low vaccine efficacy, multi- and extensively-drug resistance (MDR-XDR), extended therapy, and high TB incidence.

**Table 1:** Interaction between Mtb PAMPs and macrophage PRRs during recognition (Stamm et al., 2015; Ravesloot-Chávez et al., 2021; Tsolaki et al., 2021).

PRR Class	Category	PAMP Class	Category	Location	Induced process
**CTLRs**	MR	Glycolipids	LAM, ManLAM	Mtb cell surface	Inflammatory response inhibition (IL-10, IL-1R antagonist & type II), inhibition of IL-12 production
	DC-SIGN	Glycolipids	LAM		
	Dectin-1, 2	Unknown	Unknown		
	MINCLE	Glycolipids	TDM		
**CRs**	CR1	-	-	Mtb cell surface	Role in chronic inflammatory response
	CR3	Oligosaccharides	LAM		
	CD14	Lipoteichoic acids, peptidoglycans	LAM, Chaperonin 60.1		
**Scavenger** **receptors**	MARCO	-	LAM	Mtb cell surface	Anti-inflammatory response, autophagy activation
	SRA	Mycolic acids, diglycerides	TDM, LTA, LPS?		
	CD36	Diglycerides, lipoglycans,	LTA, ManLAM, LAM, oxidized LDL		
	MARCO	Cell wall components	LDL		
	AIM	Unknown	Unknown		
**TLRs**	TLR2	Lipoproteins, lypoglycans, secreted proteins	LAM, LM, PIM, ESAT-6, LpqH, LprG	Mtb cell surface	Pro-inflammatory cytokine production, apoptosis & autophagy induction, NF-κB activation & translocation, pro-inflammatory cytokine production (TNF, IL-1β, IL-12), IFN-γ production stimulation from surrounded immune cells (CD & T cells), enhanced antigen presentation, antimycobacterial effector mechanism promotion
	TLR2/1	Triacylated lipoproteins			
	TLR4	Cell wall lipids, glycoproteins, secreted proteins	-		
	TLR2/6	Di-acylated lipoproteins	-		
	TLR9	Nucleic acids	Mtb DNA	Phagolysosome	
**Others**	NOD2	Peptidoglycans	MDP	Macrophage cytosol	Cytokine responses to Mtb (myeloid cells)
	NLRP3	No specific and direct binding ligands	No specific and direct binding ligands		Inflammasome formation, caspase-1 activation, IL-1β production
	AIM2	Nucleic acids	dsDNA		Inflammasome pathway activation, IL-1β IL-18 production, INF1
	DNA sensings^a^ (cGAS, IFI204)	Nucleic acids	Mtb DNA		STING signaling activation, TBK activation, IRF phosphorylation, INF1 production

a These cytosolic sensors are part of the cytosolic surveillance pathway (CSP), which, when activated, causes the stimulator of IFN genes (STING) to be activated. MDP, muramyl dipeptide; ManLAM, mannose-capped lipoarabinomannan; NLR, nucleotide-binding domain and leucine-rich repeat-containing receptor; TLR, toll-like receptor; MR, mannose receptor; TDM, trehalose dimycolate; TBK, tank-binding kinase-1; IRF3, interferon-regulatory factor 3; NLRP3, NLR family pyrin domain-containing 3; cGAS, cyclic GAMP synthase; Mincle, Macrophage-Inducible C-Type Lectin.

Understanding Mtb and host macrophage coevolution will help treat and control TB. Despite significant research on diverse aspects of the Mtb-macrophage crosstalk, this subject is not entirely understood. Is it because Mtb's genetic arsenal enables it to circonstancially adapt to and survive within macrophage hostile environment? (Flentie et al., 2016). We discuss Mtb's major determinants and emerging molecular processes characterizing several stages of this interaction to elucidate essential mechanisms and reveal potential molecular targets.

## 2 Mtb effectors interacting with macrophages

Protein and lipid effectors produced by Mtb regulate the functions of macrophages and the inflammatory process (Chandra et al., 2022). The coding information of those effectors are embeded in the Mtb genome (4000 genes) (TB Database, http://tbdb.bu.edu/tbdb_sysbio/GenomesIndex.html). The mycobacterial cell envelope is a complex architectural structure consisting of a typical plasma membrane, a layer of peptidoglycans covalently attached to polysaccharides (arabinogalactans), which have their penta-arabinosyl ends esterified by mycolic acids (Brennan and Nikaido, 1995). Lipoproteins, peptidoglycans, trehalose mono- and di-mycolates, phosphatidylinositol mannosides, lipomannan, and lipoarabinomannan are all components of the Mtb cell wall that are exposed to extracellular environments (Stamm et al., 2015; Madacki et al., 2019). Other factors, such as regulatory genes (DosR, WhiB, PhoP), adenylyl cyclase protein kinases (pknG), and enzymes (Mce family proteins, for example), do not fit into the surface-exposed group (miscellaneous factors) (Madacki et al., 2019). The virulence factors mentioned above play a critical role in host-pathogen interactions, with reports stating their involvement in host-cell recognition (Vergne et al., 2014) and phagosome maturation arrest (Chatterjee et al., 1992; Fratti et al., 2003). Researchers have recently uncovered new information regarding the cell membrane's secretion mechanisms and complex mycobacterial lipids (Madacki et al., 2019). SapM and PknG are exported by the SecA2 secretion system and interfere with the acidification and maturation of the phagosome-containing Mtb (Zulauf et al., 2018). Other export routes involve the sec secretory pathway, the twin-arginine translocation (TAT) pathway, and the ESX/type VII. TAT secretion system-carried mycolyl transferases catalyze the formation of TDM by attaching mycolate residues to arabinogalactan (Belisle et al., 1997). Proteins in the PE/PPE family, so-called for the N-terminal Pro-Glu and Pro-Pro-Glu motifs they share, are released *via* the ESX-5 secretion system and may contribute to Mtb pathogenicity. Reduced PPE protein secretion, decreased cell-wall integrity, and severe attenuation, were seen in Mtb strains lacking ESX-5 (Bottai et al., 2012). However, further research is needed to reveal the underlying virulence components of the Mtb cell wall.

## 3 Mtb-macrophage interaction

### 3.1 Early Mtb-macrophage interaction

Blocking Mtb-macrophage contact and entrance into human cells can prevent TB. Mtb is an airborne pathogen that spreads from sick to healthy people. When the latter inhale Mtb-containing droplet nuclei, they reach the lungs' alveoli. Indirect (opsonization) and direct Mtb recognitions by macrophages have been reported. Indirect Mtb detection uses soluble factors (collectins, complement systems, etc.) that chemically modifiy Mtb and facilitate its internalization inside the macrophage. Recruitment of host cell molecules to Mtb cell surface has been demonstrated as well. In contrast, direct Mtb detection uses non-soluble factors that identify Mtb ligands named PAMPs.

Specific macrophage PRRs identify Mtb PAMPs on the cell surface or in the intracellular macrophage environment (phagolysosome and cytosol). Mincle and Macro receptors interact with TDM on the Mtb surface, whereas PRRs such as MR, DC-SIGN, and Dectin-2 recognize Mtb glycolipids (ManLAM). Besides, NOD2 in the cytosol detects MDP released by Mtb peptidoglycans. The TLR9 detects phagolysosomal Mtb DNA, while the ESX-1 secretion system breaks the phagosomal membrane, thus allowing the cytosolic recognition of Mtb DNA and subsequent cGAS/STING induction. Other cascade reactions activated downstream, such as phagosome biogenesis, endosomal trafficking, autophagy, or secretion of soluble factors can be benefic or detrimental for the pathogen or the macrophage (Stamm et al., 2015). For instance, indentification of the pathogenic Mtb DNA by TLR9 increased M1 macrophage-derived human monocyte antimicrobial mechanisms through phenotypic alterations, excessive TNF-α production, and autophagy activation (Ruiz et al., 2019). PRRs-activated immune signals such as PI3K, IRGM, and mTOR inhibition can also induce autophagy after Mtb infection (Deretic, 2012; Pareja and Colombo, 2013; Ruiz et al., 2019). However, more research is needed into how macrophages recognize Mtb, especially its nucleic acids.

### 3.2 Activation and polarization of macrophages

Classically activated macrophages (M1) or th1-dependent responses and alternatively activated macrophages (M2) or th2-dependent responses are two basic polarized macrophage subgroups (Gordon, 2003). M1 macrophages have metabolic reprogramming associated to Warburg effect, high NO output, high antigen presentation, and pro-inflammatory cytokine production (IL-1, IL-6, IL-12, IL-23, and CXCL9), allowing them to establish a powerful immune response against intracellular pathogens. M2 macrophages, in contrast, fight germs less effectively and repair wounds. Different stimulatory factors polarize the M1 and M2 phenotypes. TLR agonists, cytokines (IFN-γ, TNF-α, GMCSF), or chemokines can all stimulate M1 macrophages. Anti-inflammatory cytokines (IL-4, IL-10, and IL-13), glucocorticoids, immune complexes (IC), and LPS induce M2 macrophages (Wang et al., 2019).

Macrophage polarization is critical for Mtb innate immunity, and Mtb effectors are engaged in that process. For instance, the virulence-associated antigen, ESAT-6, induces the M1 phenotype in early Mtb infection and later causes the transition to M2 (Refai et al., 2018). Mtb-secreted antigens (PPE36, EspC, or Rv1987), heat-shock proteins (Hsp16.3), or genes like Rv2882c have been demonstrated to polarize macrophages *in vitro* or in mouse models, *via* the activation of crucial intracellular processes and the generation of soluble factors (Blumenthal et al., 2009; Choi et al., 2016; Guo et al., 2019; Gong et al., 2020; Zhang et al., 2020; Sha et al., 2021). However, how Mtb interferes with macrophage polarization is not holistically understood. **Table 2** summarizes recent updates of macrophage activation/polarization during Mtb infection.

**Table 2:** Recent Mtb stimuli polarizing host macrophages.

Activation/Polarization	Mtb effector	Marker	Downstream effect	Ref.
M0	PPE36	CD16, IL-6, TNFα, CXCL9, CXCL10, CCL3, CCL5	M1 polarization inhibition, cytokine storm avoidance, dampened mitochondrial activity	(Gong et al., 2020)
M1	Rv1507A	CD69, CD80, CD86, MHC I/MHCII, IL-6, IL-12, TNFα	High pro-inflammatory Th1 response, high survivability under stress conditions	(Arora et al., 2020)
M0 or M2→M1	ESAT-6	(IL-1β, IL-6, IL-12, IL-23, TNF-α, CXCL10, Cox 2, IRF5)↑, IL-10↓	Pro-inflammatory response induction at the primo-infection; promotion of bactericidal granuloma formation	(Refai et al., 2018)
M1a→M2	ESAT-6	IL-10↑, (CD80, CD86, IL6, IL-12, TNF-α, CXCL10, IRF5)↓	Anti-inflammatory response induction, formation of solid granuloma at later stage of the infection	(Refai et al., 2018)
M2	Hsp16.3	Arg-1, IL-10, TGF-β, CD206, CCRL2, CX3CR1, AKT/ERK/p38-MAPK	Latent TB infection	(Zhang et al., 2020)
M2	Rv1987	PI3K/Akt1/mTOR	Significant decrease in M2 macrophage bactericidal activity, bacterial survival	(Sha et al., 2021)

“↑” stands for “increased expression level” and “↓” stands for “decreased expression level”.

## 4 Activation of macrophage innate defenses

**Figure 1:**
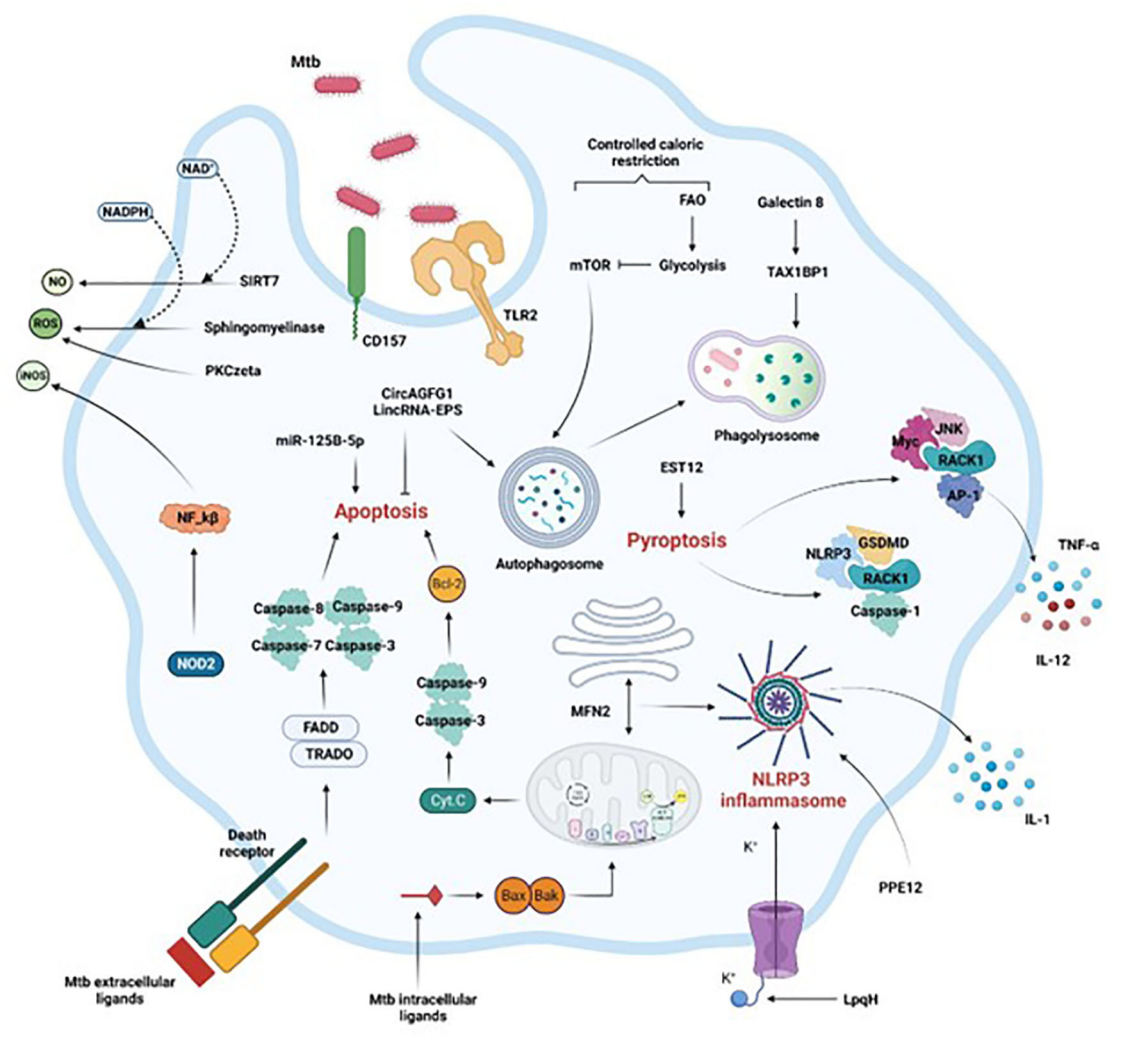
Activation of macrophage immune mechanisms to control Mtb infection. Crucial intracellular processes, such as autophagy, apoptosis, pyroptosis, and inflammation associated with the secretion of antimicrobial compounds (NO, iNOS, ROS) are induced following Mtb infection. Extrinsic or intrinsic activation of apoptosis by Mtb intracellular or extracellular effectors respectively leads to Mtb control. Mtb control is also achieved by efficient phagosome maturation and fusion to the lysosome (phagolysosome), coupled with reduced availability of calories characterized by the shift from FAO to glycolysis. Finally, the secretion of soluble factors through inflammasome and pyroptosis activation, or the production of ROS or RNS via the NF-kB pathway or through sphingomyelinases restrict the pathogen progression in the macrophage as well. FAO, fatty acid oxidation, Casp, caspase, ER, endoplasmic reticulum. Section 4 provides more details.

Critical molecular mechanisms allow host macrophages to take advantage of the crosstalk, as indicated in the sections below and in **Figure 1**.

### 4.1 Cell death activation

#### 4.1.1 Autophagy

Autophagy is an essential homeostatic process that is triggered by cellular stimuli (such as nutrient starvation) and involves nutrient regeneration, protein and organelle degradation, as well as the clearance of intracellular pathogens. Autophagic induction in macrophages is an effective mechanism to enhance intracellular killing of Mtb (Songane et al., 2012), and polymorphisms in autophagy-related genes (ATGs), especially the autophagy gene IRGM, are associated with susceptibility to TB (Intemann et al., 2009; Lu et al., 2016).

Antibacterial autophagy recruits proteins that deliver intracellular bacteria to the lysosome for degradation by ubiquitination. Sirtuin 3 (SIRT3) maintains respiratory functions. SIRT3 promotes anti-mycobacterial defenses by coordinating mitochondrial and autophagic functions (Kim et al., 2019).

Autophagy and immunometabolism drive anti-TB immunity through balanced AMPK-mTOR activation (Qu et al., 2020). This axis regulates the transcription factor TFEB, which is involved in autophagy and lysosomal biogenesis (Paik and Jo, 2020). We know little about TFEB's antimicrobial effects on autophagy and immunometabolism.

Galectins recognize Mtb-containing phagosomes and promote anti-Mtb autophagy. Galectin-8 recently interacted with the selective autophagy adapter TAX1BP1 (Deretic, 2012). BCL2-associated athanogene 2 (BAG2) reduced ER stress-induced cell apoptosis in Mtb-infected macrophages *via* autophagic flux and selective autophagy, revealing a potential host defense mechanism linking BAG2 to ER stress and autophagy during Mtb infection (Liang et al., 2020). The autophagy triggered by BAG2 required the dissociation of BECN1 and Bcl2 *via* MAPK/ERK (Liang et al., 2020).

#### 4.1.2 Apoptosis

Apoptosis is a physiological type of cell death induced by mitochondrial-mediated (intrinsically) or death receptor (extrinsically) pathways (Behar et al., 2011). Macrophage apoptosis can eliminate Mtb growth by direct antimicrobial effects or encapsulation in apoptotic bodies, which recruits new macrophages and dendritic cells (Lee et al., 2009). Activation of the Bcl-2 protein family, Bak- or Bax-mediated mitochondrial membrane damage and cytochrome C release, death receptors (Fas and TNFR), caspase activation, apoptotic body formation, efferocytosis (recognition and engulfment of apoptotic bodies by professional phagocytes *via* several cell-surface receptors), and anti-inflammatory cytokines (TGF, IL-10), can subvert intrinsic and extrinsic apoptosis to control infection to a lesser extent (Section 2). As a result, the idea of using host-directed therapies (HDTs) like inhibitors of apoptosis (IAP) proteins to manage TB infection has gained attention (Arnett and Schlesinger, 2021; Stutz et al., 2021).

#### 4.1.3 Pyroptosis

Pro-inflammatory cytokines are secreted during pyroptosis, another newly activated death process. This death process is important in inflammatory-related respiratory diseases like TB (Feng et al., 2022). Activating pyroptosis after Mtb infection may promote macrophage elimination of Mtb. Pyroptosis activation also involves the gasdermin (GSDM) family and several canonical and non-canonical inflammasome-induced pathways (caspases-1/3/6/7/GSDMB, caspase-8/GSDMC, caspase-8/GSDMD, and caspase-3/GSEME). Recently, Mtb effector EST12-induced pyroptosis activated the RACK1/NLRP3/caspase-1/GSDMD or RACK1-JNK-AP1-Myc signaling pathways to enhance the anti-mycobacterial inflammatory response (Qu et al., 2020; Wu et al., 2022). However, the precise molecular mechanisms that cause pyroptosis during TB remain enigmatic.

### 4.2 Cytotoxic molecule secretion

Macrophages produce bactericidal chemicals in response to Mtb invasion *via* a variety of ways. In macrophages, expression of the cofactor-dependent enzymatic activity mediates protective immunological responses *via* ROS and RNS. The production of ROS by acid sphingomyelinases *via* the NADPH oxidase and cathepsins enhanced infection control and BCG breakdown in macrophages, (Wu et al., 2020). Sirtuin (SIRT7) is a deacetylase that is activated by nicotinamide adenine dinucleotide (NAD^+^). Its expression in macrophages diminishes after Mtb infection. A recent study explained how the SIRT7-mediated protective mechanism leads to Mtb clearance in macrophages through NO generation and apoptosis modulation (Zhang et al., 2021). By producing ROS and NO, modified macrophage receptors produces a protective immune response. Recently, TLR2-PKCzeta-induced ROS generation *via* the CD157 receptor provided host macrophage tolerance to Mtb (Yang et al., 2019). Furthermore, following Mtb invasion, iNOS expression and NO generation in human macrophages were dependent on NOD2 expression and needed NF-κB activation (Landes et al., 2015). After Mtb infection of human macrophages, NOD2 activation increases iNOS synthesis and activity, indicating a novel molecular pathway.

### 4.3 Non-coding RNA expression

MicroRNAs (miRNAs) and long non-coding RNAs (lncRNAs) cannot encode proteins but are involved in gene regulation and Mtb infection. These entities regulate macrophage apoptosis and autophagy in active TB infection to target key host proteins for pathogen control and clearance. The circular RNA CircAGFG1 boosted autophagy and decreased apoptosis in active TB (Shi et al., 2020). Silencing miR-125b-5p could protect human macrophages from Mtb infection by inducing apoptosis and decreasing inflammation (Liu et al., 2020). This points to a specific area to focus on in the fight against Mtb.

Although the molecular link connecting lncRNAs and macrophages in TB is still obscure, lncRNA PCED1B-AS1 and lncRNA MIAT influence macrophage apoptosis and autophagy in active TB (Li et al., 2019; Jiang et al., 2021). In BCG-infected RAW264.7 macrophages, knockdown of lincRNA-EPS reduced apoptosis and increased autophagy (Ke et al., 2020). The mechanism of action of ncRNAs may offer fresh TB targets. However, these mechanisms are far to be exhaustive because novel host ncRNAs are continually discovered.

### 4.4 Inflammasome activation

The inflammasome is a multiprotein complex that includes the members of the nucleotide-binding domain and leucine-rich repeat (LRR)-containing (NLR) family and the pyrin and HIN domain (PYHIN) family. The inflammasome is important for immunity, human disease, and TB resistance. Host or pathogen components can activate the NLRP3 inflammasome (He et al., 2016). Mtb PPE13 increased IL-1β secretion *via* the NLRP3 inflammasome (Yang et al., 2020). Mtb lipoprotein LpqH has high immunogenicity and can activate the NLRP3 inflammasome through the potassium efflux route (Liu et al., 2021).

Mitochondria are essential for NLRP3 inflammasome activation as well. This importance depends on mitofusin 2 (MFN2), which participates in the creation of mitochondria-associated endoplasmic reticulum membranes (MAMs) and may be the platform for NLRP3 inflammasome production during Mtb infection. Mtb infection upregulated MFN2 expression to enhance NLRP3 inflammasome formation (Xu et al., 2020). IL-1 is a key cytokine in the immune response against TB (Silvério et al., 2021). However, there is still curiosity about how Mtb actually induces inflammation. Nevertheless, the capacity of Mtb clinical isolates to activate inflammasome and IL-1 varies. Beijing isolates, for example, showed varying effects on IL-1 and caspase-1 activation, but all clinical isolates caused lesser IL-1 release than H37Rv, indicating the involvement of NLRP3, AIM2, and an additional unknown sensor in IL-1 maturation (Subbarao et al., 2020).

### 4.5 Caloric restriction

Changes in immunometabolism are triggered by Mtb infection. Effector cell functions are susceptible to changes in the host’s nutritional status. Immune responses to infections are expensive in terms of energy expenditure, metabolic change, and food intake (Van den Bossche et al., 2017). In susceptible DBA/2 mice, pulmonary Mtb infection is mitigated by controlled caloric restriction (CR), which does not result in malnutrition. Mechanism-wise, CR caused immune cells to switch their metabolism from fatty acid oxidation (FAO) to glycolysis and reduced the mTOR activity associated with autophagy activation (Palma et al., 2021). CR is not only an unanticipated method of improving immunity to Mtb, but it may also provide a novel method of treating Mtb infection in places where TB is quickly expanding alongside overnutrition and obesity. This approach, however, has only been studied in mouse models. Nonetheless, more research is needed to determine whether or not it is applicable to human TB.

## 5 Manipulating macrophage immunity

**Figure 2:**
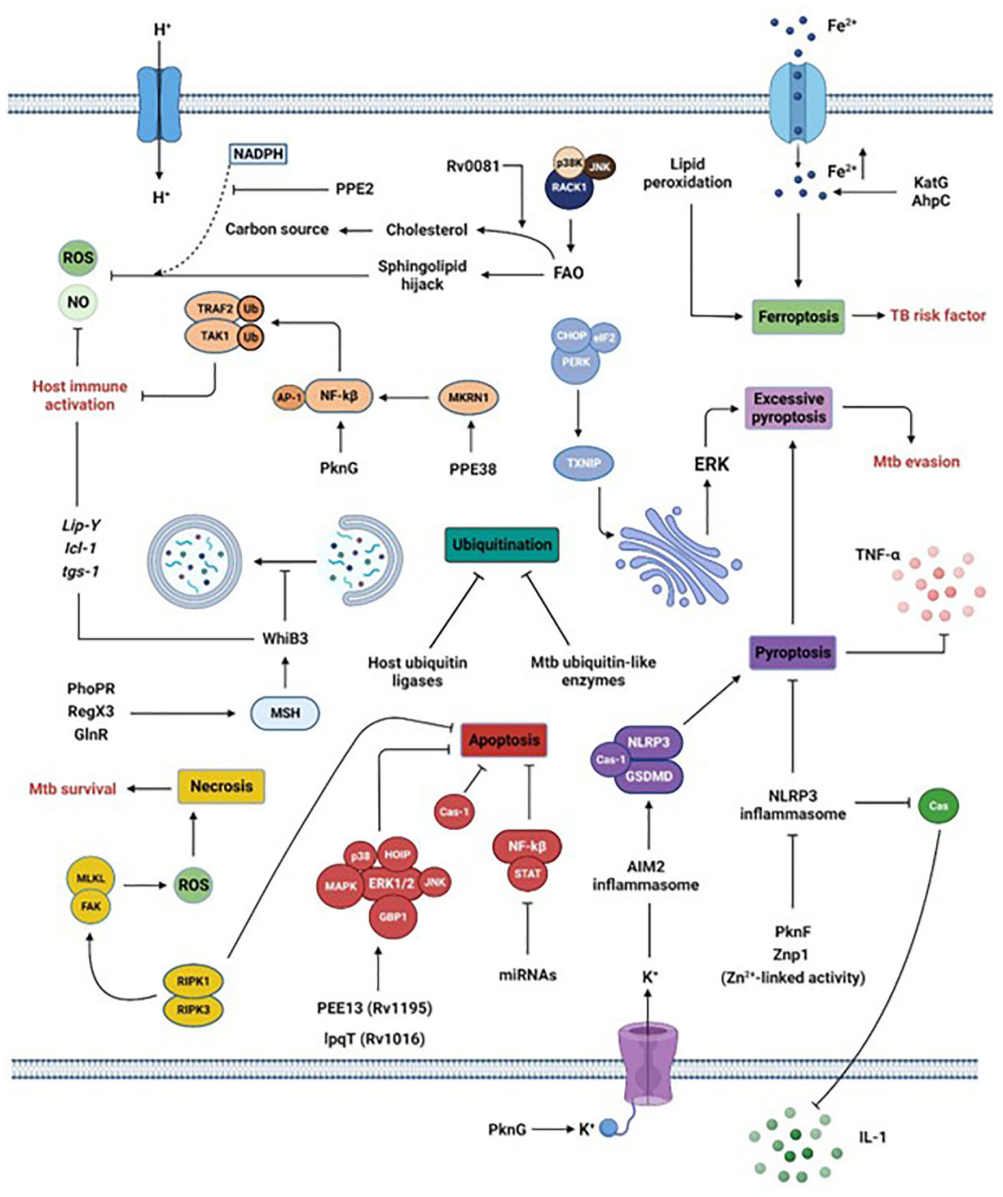
Manipulation of macrophage intracellular processes by Mtb. Via the secretion of its virulence factors, Mtb subverts crucial host processes. Mtb inhibits phagosome maturation via the MSH pathway, inhibits ubiquitination by acting as host ubiquitin-like enzymes or interfering with host ubiquitin ligases, reduces the secretion of pro-inflammatory cytokines by blocking inflammasome and pyroptosis, limits the generation of ROS or NO by interfering with sphingolipids or via the activation of the transcription factor WhiB3 or the lipid-associated gene cluster (*Lip-Y, Icl-1*, and *tgs-1*). Moreover, the promotion of ferroptosis via increased Fe^2+^ concentration or lipid peroxidation is a risk factor for individuals infected by Mtb. Finally, Mtb survival is also reached by inhibiting apoptosis while promoting necrosis. Section 5 provides more details.

Mtb can avoid, neutralize, or exploit macrophage contents for its intracellular survival. The following sections and **Figure 2** describe recent and previously unknown escape strategies.

### 5.1 Using cell deaths

Mtb hijacks host ubiquitination, autophagy, apoptosis, necrosis, pyroptosis, and ferroptosis.

#### 5.1.1 Ubiquitination

Mtb uses its effectors as ubiquitin-like enzymes or interacts with host ubiquitin ligases to reduce host ubiquitination. The protein kinase G (PknG) is a unique ubiquitin-activating enzyme (E1) and ubiquitin ligase (E3) that initiates ubiquitination and degradation of TRAF2 and TAK1 to limit host immunological activation (Wang et al., 2021). The PknG-host interaction's mechanisms are unclear. PPE38, which is encoded by the region of difference 1 (RD1), interacts with the macrophage ubiquitin ligase (E3), Makorin Ring Finger Protein 1 (MKRN1), suppressing TRAF6-driven NF-kB and AP-1 signaling, cytokines (TNF-α, IL-6), and NO production (Dou et al., 2022). This inhibition of host immunity was also reported following lysine-11-linked ubiquitination of Rv0222 by the host E3 ubiquitin ligase ANAPC2 involving SHP1 and TRAF6, or deubiquitination of TRAF6 by HAUSP after PE_PGRS38 ectopic expression in *M. smegmatis* in murine bone marrow-derived macrophages (Wang et al., 2020; Kim et al., 2022). The processes described here are previously unknown strategies used by Mtb to decrease host immunity.

#### 5.1.2 Autophagy

Mtb promotes intracellular survival by inhibiting macrophage autophagy. Many Mtb PE/PPE proteins suppress autophagy in Mtb-infected macrophages, either canonically or non-canonically (Strong et al., 2020). The enhanced intracellular survival (Eis) protein is the first Mtb effector shown to limit autophagy *via* IL-10 up-regulation and histone H3 acetylation (Shin et al., 2010; Duan et al., 2016). The strain-specific behavior of Mtb in interrupting the autophagy pathway is the blockade of autophagolysomal fusion (Ebrahimifard et al., 2022). Other key molecular mechanisms include classical Rab1A inhibition, suppression of TLR2- and MAPK-activated host functions, and decreased ERK1/2 activation (Strong et al., 2021; Strong et al., 2022). These findings demonstrate mycobacterial effectors directly interact with autophagy-initiating host proteins. PknG either dually-regulates autophagy, promotes autophagy induction by competitively binding to AKT's pleckstrin homology (PH) domain, or inhibits autophagosome maturation to restrict autophagy flux by targeting the host small GTPase RAB14 (Ge et al., 2022). Other unknown escaping strategies include induction of histone hypermethylation in ATGs (Sengupta et al., 2021), direct autophagy inhibition by RELL1 (Feng et al., 2020), miRNA inhibition of autophagy by targeting critical AGTs (ULK1, ATG7, ATG16L1, ATG4c, and NPC1) located on the lysosomal membrane during Mtb infection (Liu et al., 2020; Qu et al., 2021; Dong et al., 2022), and the implication of Mtb's sulfoglycolipids (SLs) and DIMs (Bah et al., 2020). While induction of host cell autophagy by starvation (starvation-induced autophagic elimination) is reported to kill the Mtb reference strain H37Rv *via* enhanced lysosomal delivery to mycobacterial phagosomes, its isolate Mtb Beijing strain, instead, easily resists and subdues this host blockade by exceptional upregulation of both Kxd1 and Plekhm2 genes’ expression (Laopanupong et al., 2021). KatG depletion using the CRISPR-dCas9 interference system in the Beijing isolate strain resulted in increased lysosomal delivery to its phagosome and decreased its survival upon autophagy induction by starvation (Siregar et al., 2022). This suggests the importance of KatG, Kxd1, and Plekhm2 in Mtb isolates to evade starvation-induced autophagic restriction and investigating their role in the Mtb H37Rv strain may provide interesting clues (Laopanupong et al., 2021; Siregar et al., 2022). Besides, Mtb determinants involved in this process are still widely unknown. To sum up, the mechanisms employed by Mtb to inhibit autophagy need to be understood to facilitate the design of new therapeutics or vaccines against TB.

#### 5.1.3 Apoptosis

Several Mtb pathways exploit macrophage apoptosis. Mtb thwarts or causes apoptosis by its effector proteins, regulation of host ncRNAs, and anti-apoptotic cytokine production (IL-10, IL-17A). Effector proteins of cell wall-associated glycolipids, secretion systems, serine/threonine protein kinases, heat shock, stress responses, and virulence inhibit apoptosis *via* important signaling axes (serine protease cathepsin G, GBP1, ERK1/2 signaling, IL-12p40/IL-32, IL-1/IL-6/TNF-, ROS/c-JNK, LUBAC HOIP-NF-kB, JNK/p38 MAPK) (Jayakumar et al., 2008; Danelishvili et al., 2011; Dutta et al., 2012; Halder et al., 2015; Deng et al., 2016; Wang et al., 2016; Joseph et al., 2017; Wang et al., 2017; Yang et al., 2017; Zhang et al., 2018; Long et al., 2019; Abdalla et al., 2020; Ali et al., 2020; Paik and Jo, 2020; Asaad et al., 2021). Mtb effectors such as Rv1016c (LpqH) and PE13 (Rv1195) enhance the TLR-dependent macrophage apoptosis (early infection stage) and p38/ERK/NF-kB-dependent apoptosis (late infection stage) (Aguilo et al., 2013; Li et al., 2016). Mtb determinants reduce or suppress caspases, preventing macrophage apoptosis. They also reduce anti-apoptotic IL-10 and IL-17A (Ma et al., 2019; Zhang et al., 2019; Fu et al., 2020). Mtb uses host lncRNAs less frequently than miRNAs to control apoptosis. This exploitation may include NF-κB/STAT activattion (Li et al., 2020). Further study is needed to determine how Mtb suppresses apoptosis. Collectively, the aforesaid considerations support Mtb's manipulation of host apoptosis and create a possibility for host-directed therapy targeting apoptosis for TB control.

#### 5.1.4 Necrosis

In order to promote infection development, Mtb infection inhibits apoptosis and induces necrosis by producing ROS (Zhang et al., 2005). The receptor-interacting protein kinase 3 (RIPK3) triggers numerous distinct pathways that prevent apoptosis and enhance necrosis instead, *via* ROS generation, and contribute to Mtb survival in macrophages infected with Mtb (Zhao et al., 2017). To trigger necrotic cell death, Mtb used focal adhesion kinase (FAK) in a time-dependent manner, first by exploiting RIPK1 and then, to a lesser extent, by using RIPK3-MLKL, to produce ROS (Afriyie-Asante et al., 2021).

#### 5.1.5 Pyroptosis

Additionally, Mtb escape host clearance involves pyroptosis control. The processes rely on the NLRP3/caspase-1/GSDMD axis, potassium efflux linked with Mtb PnkF expression and ROS suppression, and Mtb phagosomal inhibition of AIM2 inflammasome activation (Feng et al., 2022). Still elusive, these mechanisms may need more study. Mtb-induced excessive pyroptosis also aids in the spread of Mtb (Beckwith et al., 2020; Fu et al., 2021; Li et al., 2022). This process relies on the NLRP3/caspase-1/ GSDMD axis connected with the potassium efflux, but also strongly on the ERS induction linked with TXNIP downstream upregulation and the PERK/eIF2/CHOP axis (Gong et al., 2019; Beckwith et al., 2020; Li et al., 2022). Inhibiting pyroptosis in Mtb-infected macrophages through the PERK/eIF2/TXNIP/NLRP3/caspase-1/GSDMD axis reduces lung tissue damage and Mtb dissemination (Fu et al., 2021; Li et al., 2022).

#### 5.1.6 Ferroptosis

Ferroptosis is a new type of controlled cell death involving Fe2^+^ accumulation and lipid peroxidation. Glutathione peroxidase-4 (Gpx4) is an enzyme that plays a critical role in preventing iron-dependent lipid peroxidation-mediated cell death (ferroptosis), a process previously implicated in the necrotic pathology seen in Mtb-infected mice. Gpx4 is a crucial intracellular lipid peroxidation-detoxifying enzyme (Yant et al., 2003), and its inactivation not only participates in ferroptosis but also remains the main mechanism supporting this process (Krantz et al., 2005; Dixon et al., 2012; Yang et al., 2014). GPX4 and glutathione levels are reduced in active TB individuals, whereas those of free iron, mitochondrial superoxide, and lipid peroxidation are increased (Amaral et al., 2019; Amaral et al., 2022), suggesting a role of ferroptosis in Mtb infection. Besides, perturbed Fe2^+^ homeostasis is a TB risk factor and may serve as a TB diagnostic marker (Reddy et al., 2018; Dai et al., 2019; Meunier and Neyrolles, 2019). Ferritin deficiency-induced Fe^2+^ overload consequently promoted ROS-dependent lipid peroxidation, Mtb growth and dissemination, host death *via* accumulated lipid peroxidation, and ferroptosis of macrophages in Mtb-infected mice (Dow et al., 2021; Rastogi et al., 2021). Hence, ferroptosis seems to be closely related to pulmonary TB development and represents a potential target for pulmonary TB treatment. However, additional research is needed to elucidate the molecular mechanisms and signaling pathways underlying the connection between Mtb and ferroptosis.

### 5.2 Simultaneous manipulation of host cell deaths

Mtb successfully manipulates host immune responses by simultaneously activating more than one cell death mechanisms (aforementioned cell killing pathways). *In vitro*, human monocyte-derived macrophages (MDM)-infected virulent Mtb inhibits the apoptosis mediated by BCL-2 family molecules but, at the same time, increases the expression of molecules involved in apoptosis (BCL-2, BAX , and phosphorylated BCL-2), necroptosis (ASK1, p-38, RIPK1, RIPK3, and CASP8), and pyroptosis (NLRP3, CASP1, and IL-1β secretion) at the transcriptional and protein levels (Ramon-Luing et al., 2022). During the selective elimination of invading pathogens (xenophagy), autophagic receptor proteins (SQSTM1/p62, CALCOCO2/NDP52, and optineurin) are required, and Mtb co-opts ubiquitin effectors such as PtpA (Deretic et al., 2013; Wang et al., 2015; Chai et al., 2020). PE_PGRS41 reduces apoptosis and autophagy while boosting macrophage necrosis (Deng et al., 2017). Ferroptosis may contribute to Mtb-induced necrosis (Amaral et al., 2019). These data demonstrate Mtb's ability to control many macrophage pathways for survival.

### 5.3 Hypoxia, acidic pH, and harmful molecule neutralization

In macrophages, Mtb faces low pH, ROS, and RNS molecules. Mtb stress resistance is regulated by the cytosolic redox-sensing transcriptional regulator WhiB3. WhiB3 interacts with host gases and metabolic signals to maintain redox equilibrium (Saini et al., 2012). In addition, genes involved in lipid metabolism (*lip-Y*, *Icl-1*, and *tgs-1*) are also induced (Barrientos et al., 2022). Inhibition of the mycothiol (MSH) pathway inhibits WhiB3 activation (Mehta et al., 2016). Mtb's activation of WhiB3 in response to macrophage stressors is regulated by PhoPR, RegX3, and GlnR (Feng et al., 2018; You et al., 2019; Mahatha et al., 2020). WhiB3 activation causes redox homeostasis, down-regulation of innate immune genes, phagosomal maturation blockage, defective lysosomal trafficking, lipid anabolism regulation, virulence, and survival (Saini et al., 2012; Mehta et al., 2016; Feng et al., 2018; Mehta and Singh, 2019; You et al., 2019; Mahatha et al., 2020). However, how Mtb maintains redox balance and resistance in response to macrophage stressors is unknown. Mtb PPE2 inhibits NADPH-oxidase-mediated ROS generation (Srivastava et al., 2019). The nucleoid-associated protein M (NapM) binds to DnaA to increase Mtb's survival under stress and in macrophages (Liu et al., 2019). However, the latter is a previously undiscovered mycobacterial stress survival mechanism.

### 5.4 Granuloma nutrient utilization

Lipid metabolism affects host-pathogen interactions during Mtb infection (Davis and Ramakrishnan, 2009). Human TB develops a caseous granuloma, which implies pathogen-mediated disruption of host lipid metabolism (Kim et al., 2010). Immunohistological and biochemical investigations characterized the caseum protein and lipid species (Dawa et al., 2021). When FAO is inhibited in mouse macrophages, intracellular Mtb cannot develop (Chandra et al., 2020). Mtb can also hijack host sphingolipid balance or imitate sphingolipid enzymatic activity, which involves p38K- and JNK-dependent signaling cascades as well as surface 1-integrin and Rac1 activation (Speer et al., 2015; Li et al., 2016; Wu et al., 2018; Rolando and Buchrieser, 2019). Rv0081 promotes the use of cholesterol as the only carbon source in the granuloma (Dubey et al., 2021; Lata et al., 2022). In necroptotic granulomas, Fe2^+^ deficiency is a limiting Mtb growth factor. To avoid this difficult scenario, Mtb carefully controls the endogenous Fe^2+^ use by boosting the production of DNA repair and antioxidant activity-related proteins (KatG and AhpC) (Kurthkoti et al., 2017; Dow et al., 2021).

### 5.5 Limiting the activity of the inflammasome

Additionally, Mtb can prevent the activation of the NLRP3 inflammasome and subsequent pyroptosis in the host cell. Inhibition of the NLRP3 inflammasome linked with enhanced production of IL-1β in a caspase-1-dependent manner (Rastogi et al., 2021), is one way in which the serine/threonine kinase PknF assists Mtb in evading the host immune system. Upon inflammasome assembly, IL-1β is processed and activated, as mentioned above. A putative Zn^2+^ metalloprotease, encoded by the Mtb zmp1, inhibits inflammasome activation and IL-1β processing, thus reducing macrophage clearance of mycobacteria (Master et al., 2008). This is a previously unknown involvement for IL-1β in Mtb's modulation of the macrophage inflammasome.

### 5.6 Other Mtb evasion strategies

Modulation (upregulation) of transmembrane surface receptors (TREME2 or HRH1) can also evade macrophages (Dabla et al., 2022; Mo et al., 2022). Upregulation of these receptors activates STRING and p38MAPK-NOX2, inhibits pro-inflammatory cytokines (TNF-α, IL-1β, and ROS), and promotes anti-inflammatory cytokines (IFN-β and IL-10) (Dabla et al., 2022). Mtb effectors such as the early secreted protein target 12 (EST12), the methyltransferase (Rv1515c) encoded by the RD 6, or the dormancy regulator DosS, repress macrophage immune defenses (ROS, RNS, phagolysosomal maturation, proinflammatory response, inflammasome, antigen presentation) by activating the JAK2-STAT5a signaling pathway (Gautam et al., 2019; Yang et al., 2021; Rani et al., 2022; Yao et al., 2022). Mtb escape strategies are becoming increasingly non-negligible, and the aforementioned are recent and far to be exhaustive.

## 6 Concluding remarks and perspectives

The dialogue between Mtb and the host macrophage involves a permanent and stage-dependent interaction of Mtb's PAMPs, on one side and the macrophage's PRRs, on the other side, followed by induction of a cascade of reactions and cellular processes that activate and polarize the host macrophage. Then, the latter can control and eliminate Mtb through several processes, such as autophagy, apoptosis, inflammasome activation, ncRNA expression, phagosomal acidification, and the production of antimicrobial molecules. Unfortunately, these harsh conditions show limitations in that Mtb can subdue and manipulate them for survival. This imbalance in Mtb-macrophage crosstalks, where macrophages fail to holistically control Mtb infection, supported by the increasing discovery of previously unknown Mtb escape strategies, should be the critical point to tackle in TB control. Knowing that Mtb continually evolves and adapts, we are tempted to say that the aims assigned by the WHO to end TB may not be met by 2030. However, the fact that Mtb can simultaneously manipulate several host cell death processes may open new windows that facilitate the engineering of therapeutics with multitarget activity. Additionally, urgent attention is needed to further screen and functionally characterize Mtb's possible potential virulence factors and unveil novel mechanisms that may serve to identify new drug targets and elaborate appropriate therapeutics. Finally, it would be wise to accentuate the research by boosting host defense mechanisms and targeting critical axes of Mtb's escape mechanisms.

## Author contributions

HB and UAEM reviewed the literature, designed the figures, and wrote the manuscript. YY, JP, LL, and MW designed the tables and critically revised the manuscript. XK and HC drafted the study and revised the manuscript. All authors contributed to the article and approved the submitted version.
